# Case detection delay in leprosy: Testing tool reliability and measurement consistency in Ethiopia, Mozambique, and Tanzania

**DOI:** 10.1371/journal.pntd.0012314

**Published:** 2024-07-05

**Authors:** Ephrem Mamo, Robin van Wijk, Anne Schoenmakers, Kidist Bobosha, Mengistu Legesse, Thomas Hambridge, Kitesa Debelo, Fufa Daba, Nelly Mwageni, Abdoulaye Marega, Taye Letta, Ahmed Mohammed Eman, Banú Fumane, Helder Rassolo, Blasdus Franz Njako, Stephen E. Mshana, Jan Hendrik Richardus, Christa Kasang, Liesbeth Mieras

**Affiliations:** 1 Armauer Hansen Research Institute, Addis Ababa, Ethiopia; 2 Addis Ababa university, Addis Ababa, Ethiopia; 3 Erasmus MC, University Medical Center Rotterdam, Rotterdam, The Netherlands; 4 NLR/Leprastichting, Amsterdam, The Netherlands; 5 Deutsche Lepra- und Tuberkulosehilfe e.V. Ethiopia, Addis Ababa, Ethiopia; 6 Catholic University of Health and Allied Sciences, Mwanza, United Republic of Tanzania; 7 Lúrio University, Nampula, Mozambique; 8 Ministry of Health Ethiopia, Addis Ababa, Ethiopia; 9 NLR Mozambique, Nampula, Mozambique; 10 Deutsche Lepra- und Tuberkulosehilfe e.V. Tanzania, Dar es Salaam, United Republic of Tanzania; 11 Deutsche Lepra- und Tuberkulosehilfe e.V, Würzburg, Germany; Swiss Tropical and Public Health Institute: Schweizerisches Tropen- und Public Health-Institut, SWITZERLAND

## Abstract

**Background:**

Case detection delay (CDD) in leprosy is defined as the period between the onset of the first signs and symptoms and the time of diagnosis. A tool, consisting of a questionnaire and a detailed guide for researchers, which includes photos of typical skin signs and notes on establishing the timing of events, was developed to determine this period of delay in months in recently diagnosed leprosy patients. The aims of the study were to determine the reliability and consistency of this CDD assessment tool.

**Methods:**

This study was conducted in Ethiopia, Mozambique and Tanzania. Two types of consistency were considered: over time (test-retest reliability) and across different researchers (interrater reliability). A CDD questionnaire was administered to 167 leprosy patients who were diagnosed within 6 months prior to their inclusion. One month later, the same or another researcher re-administered the CDD questionnaire to the same patients. Both test-retest and interrater reliability were assessed using the intraclass correlation coefficient (ICC), where a value greater than or equal to 0.7 is considered acceptable.

**Results:**

In this study, 10 participants (6.0%) were under 15 years of age, and 56 (33.5%) were women. In the test-retest assessment, the mean CDD from the first and second interviews was 23.7 months (95% CI 14.4–34.8) and 24.0 months (95% CI 14.8–33.2), respectively. The ICC for test-retest reliability was 0.99 (95% CI 0.994–0.997). For the interrater reliability assessment, the first and second interviews revealed a mean CDD of 24.7 months (95% CI 18.2–31.1) and 24.6 months (95% CI 18.7–30.5), respectively, with an ICC of 0.90 (95% CI 0.85–0.94). A standard error of measurement of 0.46 months was found in the test-retest and 1.03 months in the interrater measurement. Most answers given by participants during the first and second interviews were matching (≥86%). Most non-matching answers were in the 0–2 month delay category (≥46%).

**Conclusion:**

The tool, including a questionnaire to determine the CDD of newly diagnosed leprosy patients, was validated in three African countries. The test-retest and interrater measurements demonstrated that the instrument is reliable and measures consistently. The tool can be used in routine leprosy programmes as well as in research settings.

**Trial registration:**

This trial is registered with The Netherlands Trial Register (NTR), now available via International Clinical Trial Registry Platform (ICTRP) with registration number NL7294 (NTR7503), as well as with The Pan African Clinical Trials Registry (PACTR) with registration number PACTR202303742093429.

## Author summary

Leprosy is an ancient infectious disease that still affects up to 200,000 new patients yearly worldwide, with an estimated three to four million people living with permanent disability. A delay in case detection is a major factor contributing to the development of disability. Case detection delay is defined as the interval between the onset of the disease’s initial signs and symptoms and the time of diagnosis, comprising a ‘patient delay’ and a ‘health-system delay’. The case detection delay tool was first designed in the cultural context of the East Hararghe Zone, Ethiopia, as part of the PEP4LEP project. Subsequently, it was standardised to enable cultural adaptation to other regions and countries.

The aims of this study were to determine the reliability and consistency of this case detection delay (CDD) assessment tool. The studied tool was validated in Ethiopia, Mozambique and Tanzania. The test-retest and interrater measurements demonstrated that the instrument is reliable and measures consistently. The tool can be used in routine leprosy programmes, active case detection projects, and in studies such as the PEP4LEP project. The tool is available in English, Oromiffa (Afaan Oromo), Portuguese, and Swahili via the international leprosy knowledge centre Infolep:

https://www.leprosy-information.org/resource/case-detection-delay-questionnaire

## 1 Introduction

Leprosy is a complex neglected tropical disease (NTD), caused by *Mycobacterium leprae* (*M*. *leprae*) and is primarily seen in low- and middle-income countries [1,2]. In most endemic countries, various strategies have been implemented to reduce the leprosy burden [3]. One of the relatively recently introduced strategies is single dose rifampicin (SDR) as post-exposure prophylaxis (PEP), which can reduce the risk of developing leprosy by 57% when administered to contacts of leprosy patients and is promoted by the World Health Organization (WHO) [4]. Active case detection and contact tracing is another strategy that is currently practiced in many countries [5–7]. It is a key intervention for diagnosing patients early and treating promptly to prevent disability and the further spread of the infection. The WHO’s global strategy "Towards zero leprosy (2021–2030)" includes active case detection and distribution of single dose rifampicin as post exposure prophylaxis (SDR-PEP) as one of its pillars [8].

This research is part of the Post-Exposure Prophylaxis for Leprosy (PEP4LEP) study; a cluster-randomised implementation trial in Ethiopia, Mozambique and Tanzania comparing two interventions that include contact tracing, integrated skin screening of contacts of leprosy patients, combined with SDR-PEP distribution [9]. A health centre-based approach is compared to a community skin camp approach. The rate of leprosy patients detected and case detection delay are the two primary outcomes of the PEP4LEP study [9].

*Case detection delay* is defined as the interval between the onset of the disease’s initial signs and symptoms and the time of diagnosis, comprising of a ‘patient delay’ and a ‘health-system delay’ [10]. *Patient delay* is the period between the first symptom noticed by the patient and the first visit to any formal health care provider. *Health-system delay* is the period between the patient’s first visit to any formal healthcare provider and confirmation of the diagnosis of leprosy. A delay in the detection of a leprosy diagnosis augments the transmission of *M*. *leprae* and allows disease progression. Without early intervention, patients have an increased risk of developing disabilities [11,12]. A longer case detection delay in a setting is often indicated by a high number of newly diagnosed leprosy patients with grade two (visible) disability (G2D) [11,13,14]. A *symptom* is a manifestation of disease apparent to the patient, such as a tingling sensation, while a *sign* is a manifestation of disease that is objective and observable (e.g. by a health worker), such as a skin patch [15,16]. For the readability of this article, ‘signs’ is used in the further text which could also be read as ‘signs and symptoms’.

Several studies have been conducted to determine case detection delay in different countries and contexts [10,17–22], with the mean and median values ranging from 11 to 64 months and 12 to 36 months respectively [14,23]. Previous research also identified individual, disease and community factors contributing to case detection delay including: age, sex, lack of education, lack of awareness of the early signs of leprosy, the stigma associated with the disease within communities, religious beliefs, fear of discrimination, clinical subtype and degree of disability. Lack of adequate training on leprosy and inaccessibility of health services attribute to health-system delay [11,14,24,25].

Currently, early case detection and treatment with multidrug therapy (MDT) are the mainstay for leprosy control, and assessing case detection delay is becoming a more frequently used indicator in leprosy control research [14,26–28]. A methodological tool that is adaptable to countries and cultural contexts was developed to determine leprosy diagnosis delay and has been validated and approved for use in PEP4LEP study countries, a process which was described by de Bruijne et al. [16]. Besides the case detection delay questionnaire, the tool contains a set of clinical images of leprosy signs, a body map, and a local calendar with important dates, such as holidays and religious ceremonies, to help the patient recall the time of onset of first signs [16]. Although the instrument has been piloted in several contexts, the questionnaire still required additional validation, particularly regarding reliability and measurement consistency, to ensure the tool can be used in large samples of recently diagnosed patients, in routine programmes, or in field studies. Hence, his study aimed to determine the test-retest and interrater reliability, the standard error of measurement and minimal detectable change, and the measurement consistency of the case detection delay tool in Ethiopia, Mozambique, and Tanzania.

## 2. Methods and analysis

### 2.1. Ethics statement

The PEP4LEP project is registered with The Netherlands Trial Register (NTR), now available via International Clinical Trial Registry Platform (ICTRP) with registration number NL7294 (NTR7503), as well as with The Pan African Clinical Trials Registry (PACTR) with registration number PACTR202303742093429. Ethics approval was obtained from the national researchethics review committees of Ethiopia (MoSHE/RD/4-1/1011/20), Mozambique (476/CNBS/21), and Tanzania (NIMR/HQ/R.8c/Vol.1/1530) [9]. Permission letters were obtained from the district health offices and respective health facilities if needed, and written informed consent was obtained from the study participants. The guardians of all participants below 18 years of age (co-)signed consent forms. All collected data were kept confidential and used for study purposes only.

### 2.2. Study area and period

The study was conducted from 2022 to 2023 in three Sub-Saharan African countries: Ethiopia, Mozambique, and Tanzania, which have different sociocultural contexts. In Ethiopia, the Fadis and Kurfachale districts of the East Hararghe Zone were included; in Mozambique, the Mogovolas and Murrupula districts of Nampula Province and Nampula City; and in Tanzania, Mvomero and the Morogoro district of the Morogoro Region. These locations were selected for the PEP4LEP study based on the incidence of new leprosy cases, G2D rate, the number of children diagnosed with leprosy, and reachability [29].

The three PEP4LEP countries were among the 23 global priority countries outlined in the WHO Weekly Epidemiological Report on the global leprosy situation in 2021 [29]. In 2021, Ethiopia registered 2,589 new leprosy patients, of which 11.9% had G2D. For Mozambique, the WHO report listed 3,135 new patients, of which 21.3% had G2D. In Tanzania, 1,511 new patients were registered, with 10.2% already having disabilities. It is important to mention that the 2021 data are likely still affected by the impact of the COVID-19 pandemic.

### 2.3. Study design

A cross-sectional validation study was used to assess the reliability (test-retest and interrater) and measurement consistency of the case detection delay tool for use in the PEP4LEP study.

### 2.4. Study population

One hundred sixty-seven recently diagnosed leprosy patients were interviewed using the studied, structured questionnaire to determine their case detection delay. Of these, 50% were included for test-retest and 50% for the interrater reliability assessment. Leprosy patients eligible for inclusion were diagnosed no more than six months prior to the first assessment. Patients unwilling to provide consent and those under 18 years of age who were not accompanied by a parent or legal guardian were excluded from the study.

### 2.5. Data collection instrument and procedures

The case detection delay measurement tool encompasses a questionnaire designed for the PEP4LEP study. The questionnaire was administered to leprosy patients through face-to-face interviews. It consists of ten questions aimed at collecting information on the diagnosis delay, calculated in total number of months, including the first sign or symptom of leprosy and when as well as at which body part this occurred. There are also questions on the actions taken by the patient after observing the initial signs, when these actions were taken, the first visit to a health facility, the number of times the leprosy patient visited a health facility, and when the disease was diagnosed. For consistent questionnaire administration, a "Question-by-Question Guide" was made available [16]. This guide provides explanations on the aim of the ten questions asked, ‘prompt questions’ that can be used if participants do not understand the original question, and questionnaire administration tips.

Additionally, the tool includes annexes with a set of clinical photos of leprosy signs and a local calendar with important dates, such as holidays and religious occasions, to aid patients in remembering when their first sign appeared. Previous studies showed that associating dates with significant events (religious or cultural) helps patients to remember the time of onset of their medical condition [16,30,31].

A country- and cultural context-specific version of the questionnaire was developed and validated based on the conceptual framework of Herdman *et al*. [16,32]. English, Oromiffa (Afaan Oromo), Portuguese, and Swahili versions of the case detection delay questionnaire are available via the international leprosy knowledge centre Infolep: https://www.leprosy-information.org/resource/case-detection-delay-questionnaire [33].

For interrater reliability, the case detection delay questionnaire was administered to recently diagnosed (≤6 months ago) leprosy patients and repeated one month later, with another researcher re-administering the case detection delay questionnaire to the same patients. Test-retest reliability was assessed by interviewing recently diagnosed leprosy patients using the questionnaire at two different time points, with one-month interval by the same interviewer.

The period of one month between assessments was considered long enough for the patients and the interviewers not to remember answers given during the first assessment. This one-month interval was chosen consistent with other validation studies and for operational reasons [34,35]. Data were collected by trained health and research staff: in Ethiopia and Tanzania by a research assistant and dermatologist, and in Mozambique by four trained researchers with the support of trained health workers.

### 2.6. Measurements

*Reliability* refers to the consistency of a measure; it reflects both the degree of correlation and agreement between measurements. This study looked at consistency over time (test-retest reliability) and across different researchers (interrater reliability), the level of agreement and the consistency between participants’ answers (measurement consistency) [36,37].

*Test-Retest Reliability* reflects the variation in measurements using an instrument on the same subject under the same conditions. When researchers measure a construct that they assume to be consistent across time, the scores they obtain should also be consistent across time. The time between the onset of symptoms and the moment of diagnosis does not change over time after the diagnosis has been made. The answer an individual gives today should be similar to the answer given after one month [36,37].

*Interrater Reliability* reflects the variation between two or more raters who measure the same group of subjects. When measuring case detection delay, more than two health workers conducted the interviews using the same questionnaire. Besides the fact that the case detection delay should not change over time, different health workers’ assessments should come to a similar result. If they are not the same, then those results would not be an accurately represent participants’ case detection delay [36,37].

*Agreement* expressed through the standard error of measurement (SEM), reflects the extent to which the scores of repeated measures are close to each other, spread around a ‘true’ score (absolute measurement error) [38,39].

*Measurement Consistency* of the answers given by participants reflects the extent to which a participant provides the same information when being interviewed again after one month. It is expressed as the percentage of matching answers and the percentage of non-matching answers, among the answers that were expressed in months, in the category of small differences of 0–2 months.

### 2.7. Statistical analysis

The data were checked, cleaned and analysed using SPSS version 25. Data were summarised in terms of frequency, proportion, measure of central tendency and dispersion. The distribution of the dataset was evaluated primarily by making histograms and then by the Kolmogorov-Smirnov test. The mean and 95% confidence interval (CI) were used to describe diagnosis delay. Both test-retest reliability and interrater reliability were determined using the intraclass correlation coefficient (ICC) value, where a value greater than or equal to 0.7 is considered acceptable [40–42]. A significance level of 5% was used to assess whether there were differences in the responses obtained in the test and retest [43]. Agreement is tested by calculating: [1] the standard error of measurement (SEM) using the formula SEM = standard deviation (SD) of the first test * (√(1-ICC), [2] the minimal detectable change (MDC _individual_) using the formula 1.96 * √2 * SEM, and [3] minimal detectable change group (MDC _group_) by dividing the minimal detectable change individual by √n [38,39,44]. The frequency of matching and non-matching responses between the two consecutive administrations of the case detection delay questionnaire one month apart was also measured. The time intervals of one month between the interviews were taken into account when assessing whether they matched or not.

## 3. Results

### 3.1. Characteristics of the study participants

A total of 167 recently diagnosed leprosy patients were involved in this study after excluding questionnaires with missing values. Among them, 50 (29.9%) were from Ethiopia, 77 (46.1%) from Mozambique, and 40 (24.0%) from Tanzania. Ten (6.0%) were children under 15 years of age, and the mean age of the participants was 39.0 ±16.2 years. Additionally, 56 (33.5%) were women (Table 1).

**Table 1:** Overview of the study participants in the three countries.

Variable	Frequency	Percentage
**Countries** Ethiopia Mozambique Tanzania	50 77 40	29.9% 46.1% 24.0%
**Sex** Male Female	111 56	66.5% 33.5%
**Age group** <15 years 16–30 years	10 51	6.0% 30.5%
31–45 years	50	29.9%
46–60 years	36	21.6%
>60 years	20	12.0%

### 3.2. Test-retest reliability

As shown in Table 2, it was observed that the patients included in Mozambique had a longer mean delay of 32.0 months (95% CI 14.0–50.0) in both the first and second tests, compared to Ethiopia and Tanzania. However, there was no significant mean difference in the subgroups (sex, age, and countries; *p*>0.05). The overall mean case detection delay of the first and second interviews was 23.7 months (95% CI 14.4–34.8) and 24.0 months (95% CI 14.8–33.2), respectively. A high degree of intraclass correlation was found between the first and second tests, with an ICC of 0.99 (95% CI 0.994–0.997; *p*<0.001).

**Table 2:** Test-retest assessment of a questionnaire to determine case detection delay.

Variable	First test				Second test		
	n (%)	Mean case detection delay (months)	95% CI for mean		Mean case detection delay (months)	95% CI for mean	
			Lower bound	Upper bound		Lower bound	Upper Lower
**Countries**							
Ethiopia	25 (31.2%)	13.4	8.6	18.2	13.5	8.3	18.7
Mozambique	40 (50.0%)	32.0	14.0	50.0	32.0	14.1	50.1
Tanzania	15 (18.8%)	18.7	12.5	24.8	20.1	12.9	27.3
**Sex**							
Male	51 (63.8%)	19.9	13.4	26.5	20.1	13.3	26.8
Female	29 (36.2%)	30.3	7.0	53.5	30.9	7.7	54.2
**Age group**							
<15 years	6 (7.5%)	11.5	5.5	17.5	11.2	5.0	17.3
16–30 years	23 (28.8%)	31.5	4.5	58.3	31.7	4.8	58.6
31–45 years	20 (25.0%)	26.9	5.8	48.1	28.0	6.9	49.1
46–60 years	22 (27.5%)	16.1	10.8	21.5	15.9	9.8	22.0
>60 years	9 (11.2%)	23.4	7.8	39.0	23.9	8.1	39.7
Total	80 (100%)	23.7	14.4	34.8	24.0	14.8	33.2

ICC: 0.99 (95% CI = 0.994–0.997)

Abbreviations: CI: confidence interval, ICC: intraclass correlation coefficient, n: number

### 3.3. Interrater reliability

The case detection delay measurements by different pairs of raters are shown in Table 3.

**Table 3:** Interrater reliability in administering the questionnaire to determine the case detection delay.

Variable	Rater A				Rater B		
	n (%)	Mean case detection delay (months)	95% CI for mean		Mean case detection delay (months)	95% CI for mean	
			Lower bound	Upper bound		Lower bound	Upper bound
Countries							
Ethiopia	25 (28.7%)	17.7	3.9	31.4	17.5	8.16	26.8
Mozambique	37 (42.6%)	21.7	16.0	27.3	21.8	16.2	27.5
Tanzania	25 (28.7%)	36.0	19.9	52.2	35.8	19.4	52.3
**Sex**							
Male	60 (69.0%)	27.8	18.8	36.8	27.2	19.1	35.4
Female	27 (31.0%)	17.7	12.3	23.1	18.8	13.3	24.3
**Age group**							
<15 years	4 (4.6%)	20.5	35.2	76.2	21.0	30.9	72.9
≥15 years	83 (95.4%)	24.8	18.3	31.4	24.8	18.8	30.8
Total	87 (100%)	24.7	18.2	31.1	24.6	18.7	30.5

ICC = 0.90 (95% CI 0.85–0.94)

Abbreviations: CI: confidence interval, ICC: intraclass correlation coefficient, n: number

There is no difference in overall mean detection delay between the two raters. The first interviews resulted in a mean case detection delay of 24.7 months (95% CI 18.2–31.1), while the second interviews resulted in a mean case detection delay of 24.6 months (95% CI 18.7–30.5). The ICC between the raters was high, with a value of 0.90 (95% CI 0.85–0.94; *p*<0.001).

### 3.4. Agreement

The standard error of measurement was calculated to be 0.46 for test-retest and 1.03 for interrater testing (see Table 4). The MDC for individual assessment was 1.15 and 2.84 months, and the MDC for group assessment was 0.13 and 0.31 months respectively.

**Table 4:** Test-retest and interrater agreement to determine the case detection delay.

	SD mean first test	SEM _agreement_	MDC _individual_	MDC _group_
Test-retest	4.61	0.46	1.15	0.13
Interrater	3.24	1.03	2.84	0.31

Abbreviations: MDC: minimal detectable change, SD: standard deviation, SEM: standard error of measurement

### 3.5. Measurement consistency of participants’ answers

The frequency of matching and non-matching responses between the two consecutive administrations of the case detection delay questionnaire, taken one month apart, is shown in Table 5. When determining whether the answers matched, the time difference between the answers was taken into account. In response to question 1, "Which sign was it that you noticed first?", the answer was the same for 154 patients for the first and second time; there were 13 non-matching replies. For question 2, "In what year did you notice this first sign or symptom of your disease?", 146 patients gave the same responses, 21 replies were unmatched. For question 3, "To specify, how many months ago did you notice the first signs or symptoms of your disease?", 147 had matching responses, while 20 did not. The unmatched responses for question 3 were further categorised into different groups: small differences (≤2 months), intermediated differences (≤4 months), large differences (>4 months). The majority (55.0%) were in the category of small differences, with an average difference of 1.9 months.

**Table 5:** Answer consistency assessment of the questionnaire to determine case detection delay.

n	Question	Matching (n)	Non-matching (n)	Non-matching frequency (n)		
				≤2 months	2–4 months	≥4 months
1	Which sign was it that you noticed first?	154	13	n/a	n/a	n/a
2	In what year did you notice this first sign or symptom of your disease?	146	21	n/a	n/a	n/a
3	To specify, how many months ago did you notice the first signs or symptoms of your disease?	147	20	11 (55.0%)	4 (20.0%)	5 (25.0%)
4a.*♦	Can you tell me how your disease developed from the signs and symptoms you have noticed and how long you have had these signs?					
	Skin patch	92	9	4	2	3
	Loss of sensation	35	5	3	-	2
	Numbness	6	-	-	-	-
	Nodule	5	3	3	-	-
	Claw hand	2	-	-	-	-
	Wound	7	3	3	-	-
5♦	Which steps were taken after you noticed the first signs or symptoms and when were these steps taken?					
	Visiting a health facility	88	10	5	2	3
	Visiting traditional healer	26	4	2	2	
	No action taken	20	7	4	1	2
	Self-treatment with remedies	10	2	-	-	2
6	When was your first visit to a health facility?	145	22	10 (45.5%)	5 (22.7%)	7 (31.8%)
7	How many times did you visit a health facility before you received your diagnosis?	150	17	n/a	n/a	n/a
8	When did you receive your diagnosis of leprosy?	145	22	10 (45.5%)	5 (22.5%)	7 (31.8%)
9	As the next question is very important, I would like to ask you again: if you think about the signs of your disease and the questions that were asked before, how long ago did the first signs of your disease show?	147	20	11 (55.0%)	4 (20.0%)	5 (25.0%)
10	Start of the first signs and symptoms of leprosy	147	20	11 (55.0%)	4 (20.0%)	5 (25.0%)

*Question 4b is about indicating the first sign/symptom on a body map and therefore not included in this table.

♦ Names signs/symptoms and named actions taken were listed by the included participants.

Abbreviations: n: number, n/a: not applicable

The answers given to question 5, "Which steps were taken after you noticed the first signs or symptoms and when were these steps taken?", were consistent for 144 patients, and inconsistent for 23 patients. In response to question 6, "When was your first visit to a health facility?", the answer was the same for 145, and 22 replies were unmatched, where most (45.5%) of the unmatched responses had a less than 2 months difference. Similarly, for question 8, "When did you receive your diagnosis of leprosy?", the answers were consistent for 145 patients, and inconsistent for 22 patients. Of the inconsistent responses, 45.5% were based on small differences, with an average difference of 2.8 months.

## 4. Discussion

This study assessed the validity of the case detection delay tool in Ethiopia, Mozambique and Tanzania, demonstrating that the tool consistently measured case detection delay. The pilot, carried out in the same study areas, also demonstrated the tool’s consistency and adaptability to various cultural contexts [16]. A study by Dharmawan et al. in Indonesia using the same leprosy case detection delay questionnaire, which was translated, culturally adapted and evaluated, also confirmed that the tool provides consistent answers [45].

Timely diagnosis with adequate treatment and follow-up minimises the risk of leprosy patients developing complications and permanent disabilities. Several studies have measured case detection delay in leprosy and identified factors affecting diagnosis delay [18,20–22,25,46–49]. According to the most recent WHO “Weekly epidemiological record” on leprosy, African countries account for half of all newly diagnosed children with leprosy-related disabilities at the time of diagnosis, highlighting the importance of understanding context specific reasons for case detection delay [50]. Similarly, Dharmawan et al. emphasises the importance of understanding of context-specific case detection delay [45]. However, a major challenge in some studies was the lack of standardised tools to assess case detection delay, indicating the importance of a reliable and validated case detection delay assessment tool [16,47].

The PEP4LEP case detection delay tool was designed to help patients to better recall specific events, such as the time of the first sign or symptom, when leprosy was first considered, or the first place visited for medical help. These events may be hard to recall if the signs were/seemed minor at first and/or had been present for months or even years [30,49,51,52]. Based on the work of de Bruijne et al. and the PEP4LEP study protocol, we administered the questionnaire including only patients who had been diagnosed up to six months prior to inclusion [9,16]. A local calendar was incorporated as a supplement, to help overcome memory loss, making the tool more consistent and reliable [16,53,54].

A key strength of the study was the use of skilled data collectors, trained to use the questionnaire, and the “Question-by-Question Guide” for administering the questionnaire. To overcome memory loss and gain clarity on early signs of the disease, we provided recently diagnosed leprosy patients with, in addition to the local calendar, photos of various leprosy signs and a body map to indicate the location of their signs and symptoms. A limitation of the study was the possibility that participants might recall answers from the first interview and respond from memory. Similarly, raters might remember answers from the first interview when conducting the second interview [55]. This may partly explain why some of the findings of the first and second interviews are so similar; which may not solely be the effect of the tool’s quality. To minimise this effect, a one-month interval between interviews was set for both the test-retest and interrater reliability studies. The duration between the test moments also influences consistency;, a longer duration is likely to result in lower consistency [34,56]. The one-month duration in this study was chosen based on other validation studies as well as for operational reasons, ensuring patients could still be found for a second interview round [34,35].

We have demonstrated that the case detection delay tool is reliable for determining the duration of case detection delay in the context of the three sub-Sahara African countries. The study showed that the tool can be used in different contexts to compare case detection delay across patient cohorts. It can contribute to the planning and monitoring of future interventions to diagnose and treat patients early and prevent leprosy-related disability; ultimately helping to halt leprosy transmission in communities.

## 5. Conclusion

The studied tool, including a questionnaire, to determine the case detection delay of newly diagnosed leprosy patients was validated in Ethiopia, Mozambique and Tanzania. The test-retest and interrater measurements demonstrated that the instrument is reliable and measures consistently. The tool can be used in routine leprosy programmes, active case detection projects and in research projects such as PEP4LEP. This study recommends further adaption and validation of the case detection delay tool in other countries, to make this tool applicable across multiple contexts.
